# Supplementary material for: Acidic pH can attenuate immune killing through inactivation of perforin
Source: EMBO Rep. 2025 Jan 9;26(4):929–47. doi: 10.1038/s44319-024-00365-6 (PMC11850619; doi:10.1038/s44319-024-00365-6)
Supplement: Supplementary file 1 — Appendix [file 44319_2024_365_MOESM1_ESM.pdf]

Appendix

Table of Contents

Appendix Figures 2

Appendix Figure S1: pH stable cell media tests for cell viability and pH stability. 2

Appendix Figure S2: Evaluation of SRBC lysis assays using turbidity in MMT buffer. 3

Appendix Figure S3: WT-PRF binds a supported lipid bilayer at pH 5 as detected by AFM and TMH1-PRF has no lytic activity in the absence of Ca^{2+}. 4

Appendix Figure S4: FACS strategy for CAR T cells. 5

Appendix Figure S5: Analysis strategies for flow cytometry data. 6

Appendix Figure S6: Anti-human perforin antibody reacts with disulphide-locked TMH1-PRF. 7

Appendix Figure S7: Flow-cytometry based phenotype analysis of NK-92 for cell line verification. 7

Appendix Tables 8

Appendix Table S1: Osmolarity measurements of pH stable media compared to standard cell media. 8

Appendix Table S2: STR results and analysis for cell line identification. 8

Appendix Figures

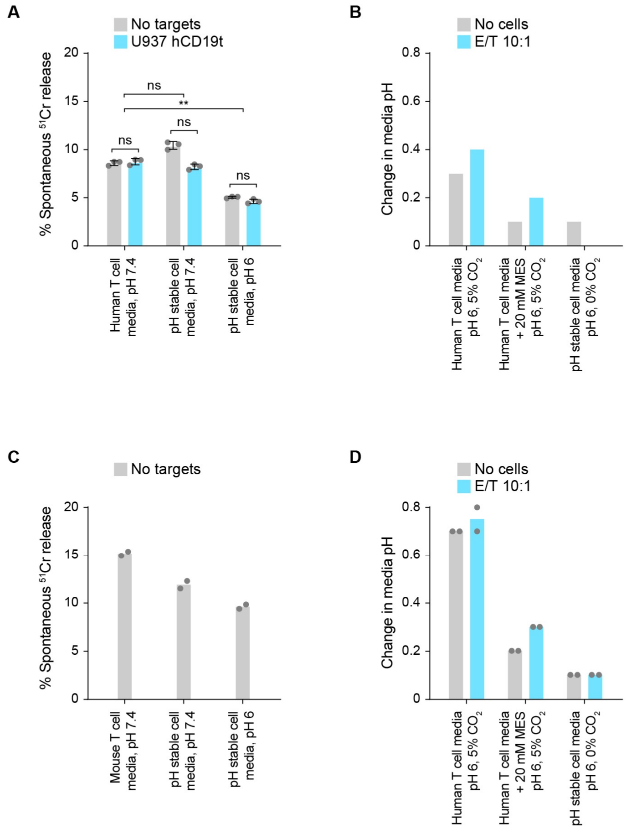

Appendix Figure S1: pH stable cell media tests for cell viability and pH stability.

(A) To confirm that effector cell viability in pH stable media is comparable to human T cell media, spontaneous ^{51}Cr release of anti-hCD19 CAR T cells was assessed after 4 h at 37 °C, 0% CO_2 in pH stable cell media at pH 7.4 and pH 6, or 5% CO_2 in human T cell media at pH 7.4. Cell density was at $5*10^5$ CAR T cells/mL, with or without $5*10^4$ U937 hCD19t target cells/mL.

(B) To assess the stability of pH 6 in different media, we measured the pH before and after 4 h at 37 °C and plotted the increase in pH. Media pH stability was assessed either without cells or in the presence of $5*10^5$ CAR T effectors and $5*10^4$ U937 hCD19t targets (10:1 E/T ratio).

(C) Analogous measurement of cell viability as in A) using mouse derived OTI effectors.

(D) Analogous measurement of pH stability as in B) using either no cells or OTI effectors and SIINFEKL pulsed EL4 target cells. For measurements at high CO_2, the pH probe was locked inside an incubator with the samples. Overall, pH stable media supports similar effector cell viability as a standard culture media and have a stable pH within 0.1 pH increment under the conditions used in our assays.

Data information: All pH values were rounded to the closest 0.1 increment. Measurements represents mean ± SD of n = 3 (A) or the mean of n = 2 (C, D) technical replicate samples (using either 10% v/v fetal bovine serum or 0.1% w/w bovine serum albumin), or a single experiment, n = 1 (B). In (A), Kolmogorov-Smirnov tests were used for statistical analysis, ** $p = 2.2*10^{-3}$, ns, not significant, p = 0.6 (Human T cell media, pH 7.4), p = 0.1 (pH stable cell media, pH 7.4), p = 0.1 (pH stable cell media, pH 6), p = 0.5 (Human T cell media, pH 7.4 pooled vs. pH stable cell media, pH 7.4 pooled).

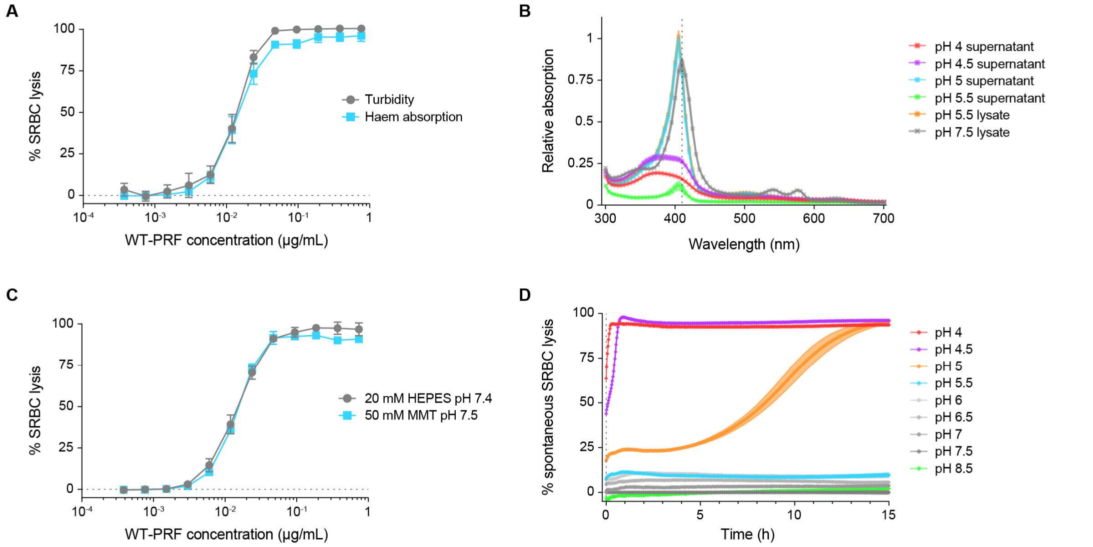

Appendix Figure S2: Evaluation of SRBC lysis assays using turbidity in MMT buffer.

(A) SRBC lysis curves produced by WT-PRF show the same concentration dependent behaviour when using haem release or change in turbidity to detect lysis.

(B) Absorption spectra of haem at different pH, recorded between 300-700 nm in 5 nm increments of supernatant or pellets lysed in water as denoted, after 15 h incubation at 37 °C. Acidification produced a shift in the haem absorption peak to lower wavelengths (410 to 405 nm) accompanied by a ~20% increase of absorption at pH 5.5 and pH 5, and the loss of a defined peak at pH 4.5 and pH 4. This renders haem absorption less suitable to measure perforin dependent lysis at acidic pH, compared to turbidity-based measurements.

(C) Using turbidity change as detection method, WT-PRF induces identical concentration dependent SRBC lysis in HEPES and in MMT based buffers at neutral pH.

(D) In a timelapse measurement of spontaneous SRBC lysis at different pH using turbidity change as detection method, SRBCs remained stable for 15 h at pH 5.5-8.5 and 37 °C, and spontaneously lysed at pH 4-5.

Data information: the data shown in (A, C) was collected from n = 3 technical replicates and depicts mean ± SE. The data shown in (B, D) was collected from n = 3 technical replicates and shows mean ± SD as coloured background band.

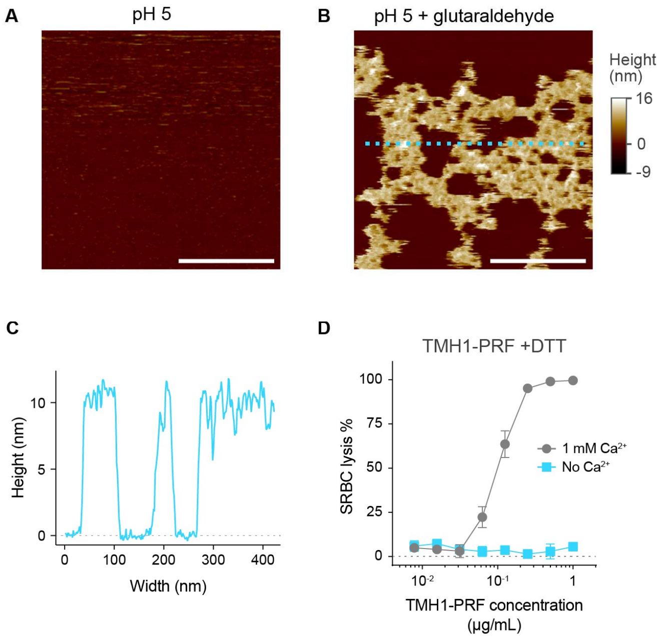

Appendix Figure S3: WT-PRF binds a supported lipid bilayer at pH 5 as detected by AFM and TMH1-PRF has no lytic activity in the absence of Ca^{2+}.

(A) At pH 5, no WT-PRF pores are static and readily detected by AFM in the supported lipid bilayer. However, potentially bound protein that is not membrane inserted is not readily resolved by AFM due to its high lateral mobility (Leung et al, 2017), and the membrane may appear devoid of protein. Image for illustrative purpose re-used from **Figure 3B**.

(B) To assess whether WT-PRF binds the lipid membrane at pH 5 but is too mobile, we added glutaraldehyde to cross-link bound protein into larger plaques and slowing it sufficiently to be resolved by AFM. At pH 5, we indeed observe the formation of plaques after addition of glutaraldehyde, indicating that WT-PRF binds at pH 5 without forming pores. The dashed blue line indicates the position used for a cross-section shown in (C).

(C) These plaques reach 11 nm in height, corresponding to the height of an upstanding perforin monomer.

(D) To test whether any calcium independent binding of TMH1-PRF, visible at neutral pH in **Figure 4A,** might lead to the formation of transmembrane pores, we tested the lytic activity of the protein in the presence and absence of calcium. The disulphide locked mutant TMH1-PRF was titrated and incubated with SRBCs in MMT pH 7.4 in the presence of 4 mM of the reducing agent DTT, which reduces the TMH1 disulphide bond and restores lytic function, and with or without 1 mM Ca^{2+}. Lysis was detected as a measure of change in turbidity and plotted against TMH1-PRF concentration, showing lytic activity exclusively in the presence of Ca^{2+}.

Data information: (A, B) scale bars, 200 nm. In (D), data represents mean ± SE from n = 3 technical replicates.

A Anti-hCD19 CAR-T (myc-tag)

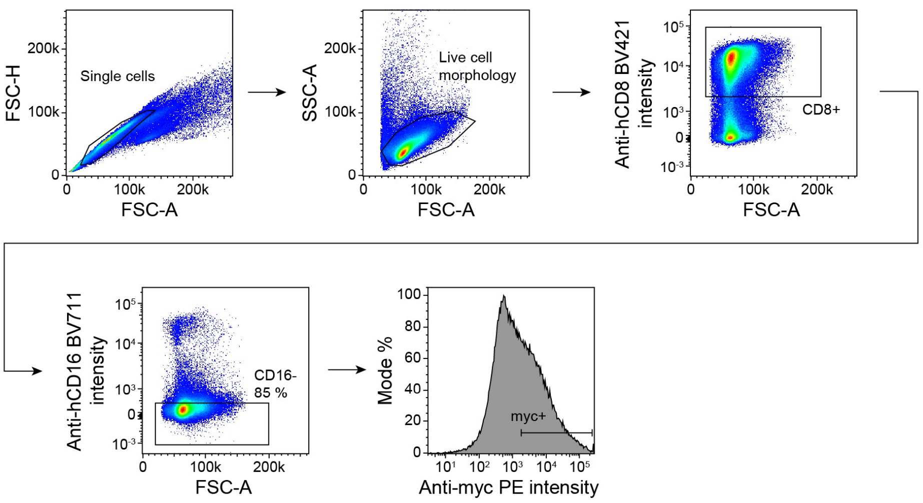

B Anti-hCD19 CAR-T (flag-tag)

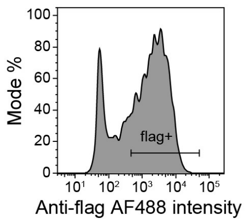

Appendix Figure S4: FACS strategy for CAR T cells.

(A) Gating strategy to sort for retroviral vector transduced CAR T cells expressing an anti-hCD19 CAR with a myc-tag.

(B) Gating strategy to sort for lentiviral vector transduced CAR T cells was done as in (A) except for using a flag-tag, with an example gating shown.

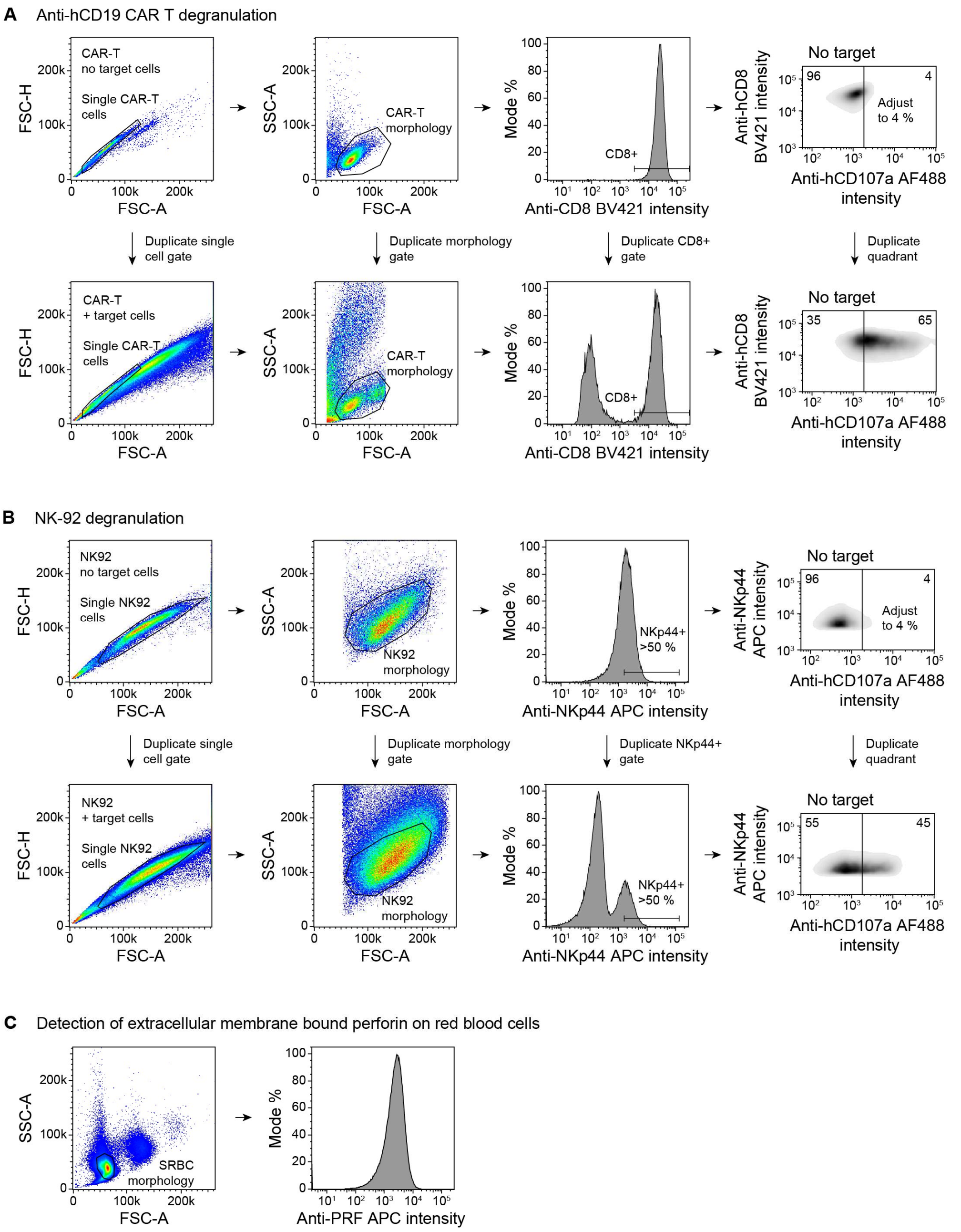

Appendix Figure S5: Analysis strategies for flow cytometry data.

(A) Analysis strategy to evaluate degranulation of CAR T cells. Where applicable and in addition to the shown gating strategy, CAR T effectors have also been gated for flag or myc positivity to distinguish between CAR positive and non-transduced CTLs (see **Appendix Figure S4**).

(B) as in (A) for NK-92 cells.

(C) Morphology gating applied to SRBCs to evaluate perforin binding.

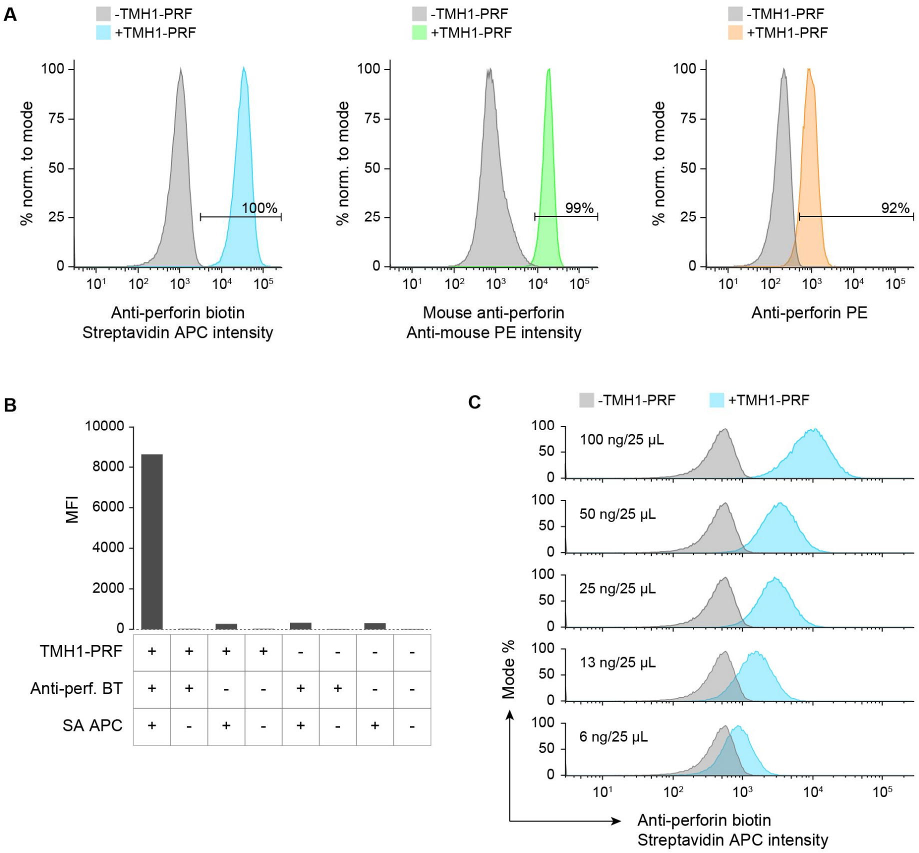

Appendix Figure S6: Anti-human perforin antibody reacts with disulphide-locked TMH1-PRF.

(A) Three different conjugates of anti-human perforin antibodies (clone: δG9) from three different manufacturers (see Reagents and Tools Table) produce fluorescent signals when TMH1-PRF is present on K562 cells, as detected by flow cytometry. All samples contained 10^5 cells and were incubated with 100 ng TMH1-PRF for 15 min on ice and subsequently labelled in 1/25 antibody dilutions in 25 µL of media for 30 min on ice. Where applicable, primary and secondary stains were incubated sequentially.

(B) Using the biotinylated antibody shown in (A), a substantial fluorescence signal was only obtained when TMH1-PRF, the biotinylated anti-perforin antibody (anti-perf. BT), and the streptavidin conjugated fluorophore (SA APC) were present.

(C) When antibody concentration and cell number were fixed, the brightness of the fluorescent signal depended on the concentration of TMH1-PRF, indicated in each panel.

Data information: In (B) data is presented from a single experiment, n = 1.

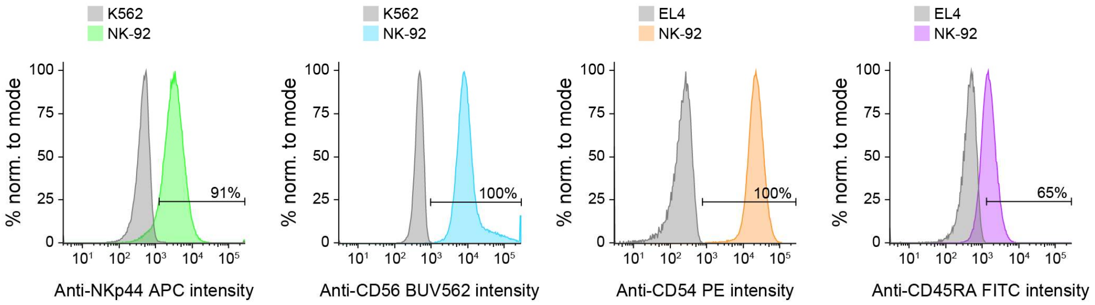

Appendix Figure S7: Flow-cytometry based phenotype analysis of NK-92 for cell line verification.

Appendix Tables

Appendix Table S1: Osmolarity measurements of pH stable media compared to standard cell media.

Medium	Osmolarity (mOsmol/kg)*
pH stable media pH 7.4	317±1
pH stable media pH 6	304±2
Human T cell medium	289±3
DMEM, 1X Glutamax, 10% FCS	347±2
20 mM MES in Milli-Q H_2O	26±16
20 mM HEPES in Milli-Q H_2O	20±6

* Mean ± standard deviation from three technical replicates

Appendix Table S2: STR results and analysis for cell line identification.

Cell line	Amelogenin	CSF1PO	D13S317	D16S539	D21S11	D5S818	D7S820	TH01	TPOX	vWA	STR reference profile	Tanabe match
K562	x, x	9, 10	8, 8	11, 12	29, 30, 31	11, 12	9, 11	9.3, 9.3	8, 9	16, 16	K-562 ATCC CCL-243	98%
U937	X, X	12, 12	10, 12	12, 12	27, 29	12, 12	9, 11	6, 9.3	8, 11	14, 15	U937 ATCC CRL-1593.2	85%
