# Supplementary figures and images for: Acidic pH can attenuate immune killing through inactivation of perforin
Source: EMBO Rep. 2025 Jan 9;26(4):929–47. doi: 10.1038/s44319-024-00365-6 (PMC11850619; doi:10.1038/s44319-024-00365-6)

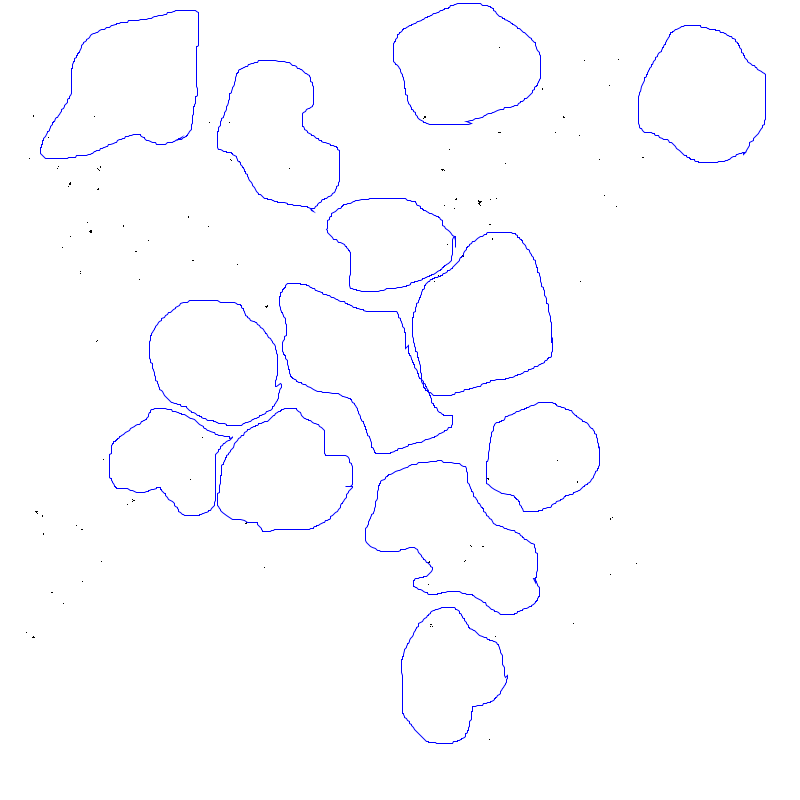

Supplement: Supplementary file 4 — Source data Fig. 2 [file 44319_2024_365_MOESM4_ESM.zip › Figure 2/2B/2B, Empty, ALFA-PRF.tif]

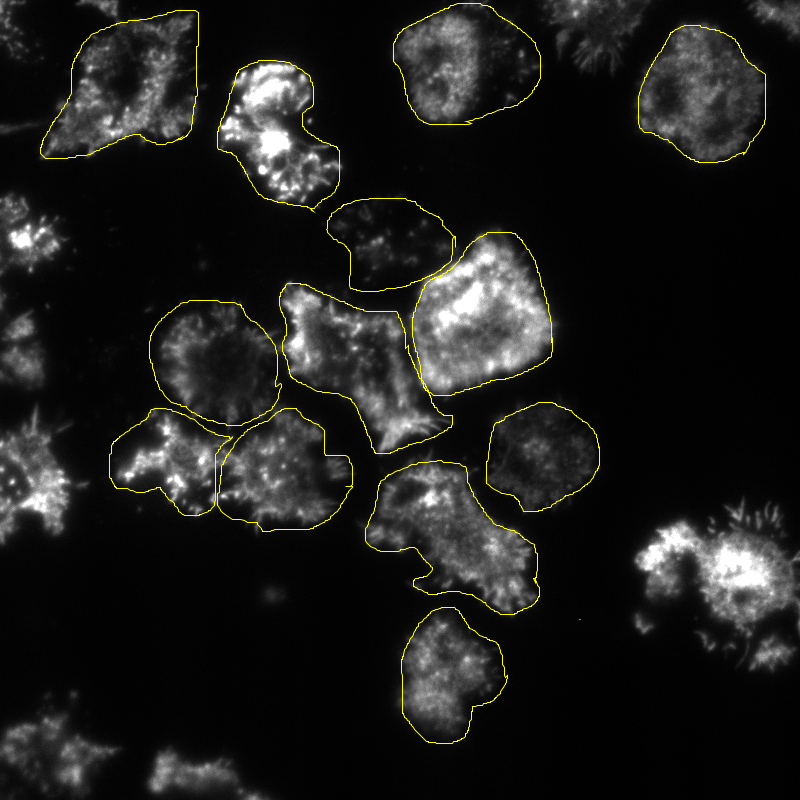

Supplement: Supplementary file 4 — Source data Fig. 2 [file 44319_2024_365_MOESM4_ESM.zip › Figure 2/2B/2B, Empty, F-actin.tif]

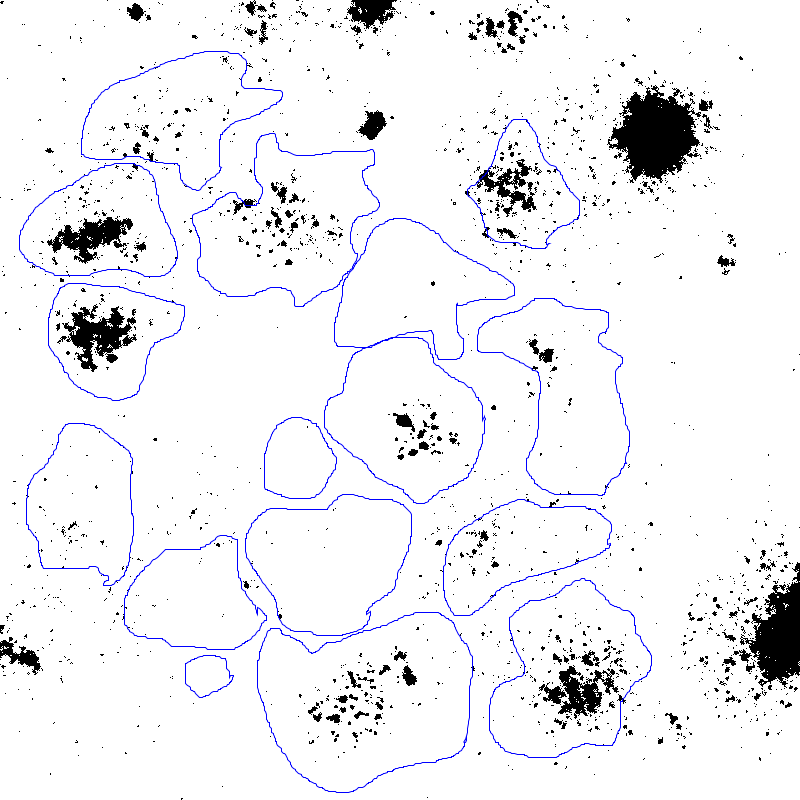

Supplement: Supplementary file 4 — Source data Fig. 2 [file 44319_2024_365_MOESM4_ESM.zip › Figure 2/2B/2B, pH 6, ALFA-PRF.tif]

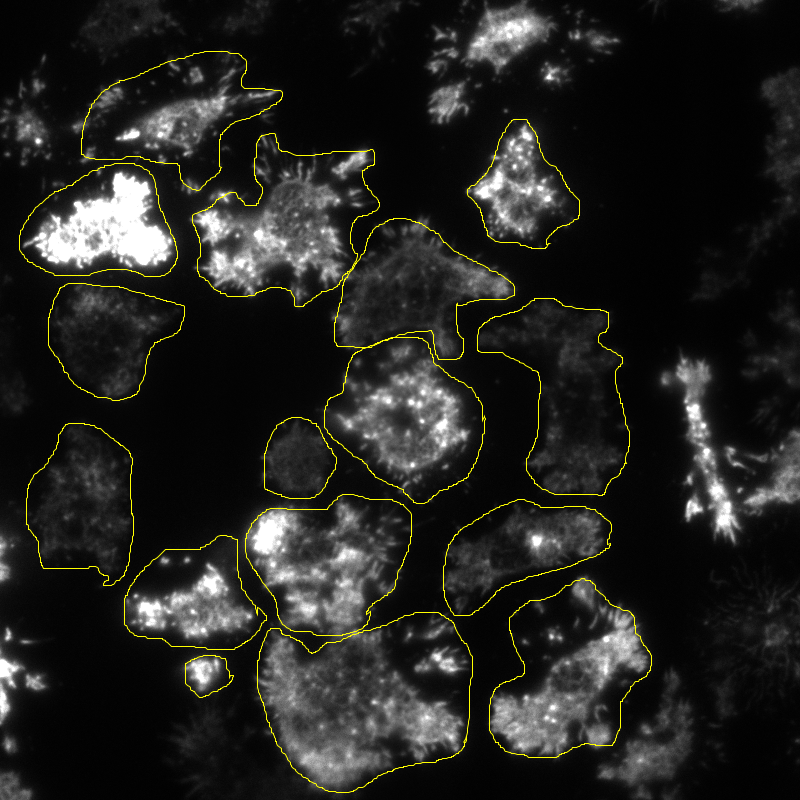

Supplement: Supplementary file 4 — Source data Fig. 2 [file 44319_2024_365_MOESM4_ESM.zip › Figure 2/2B/2B, pH 6, F-actin.tif]

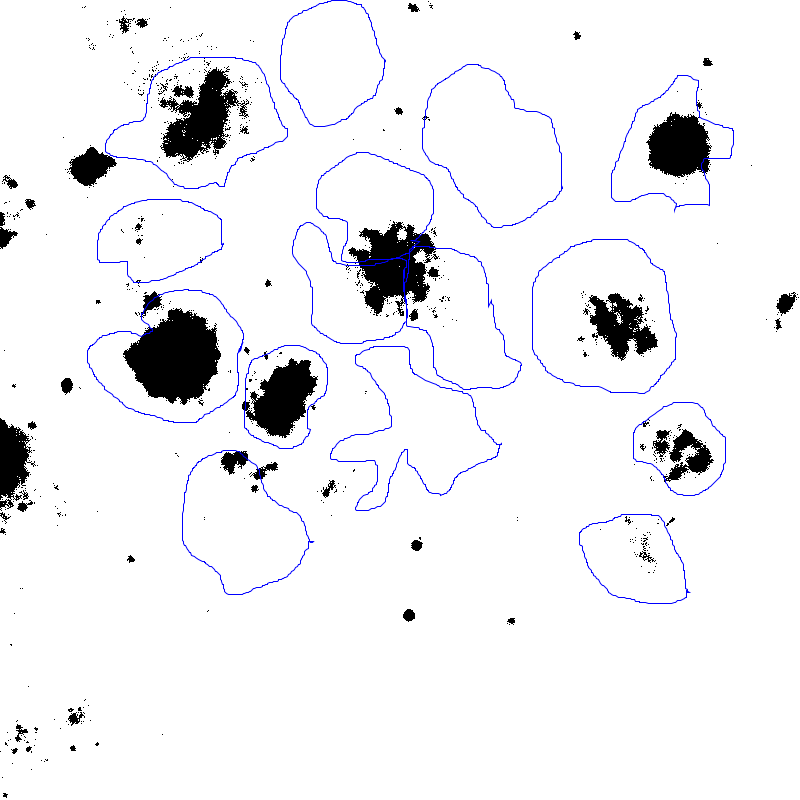

Supplement: Supplementary file 4 — Source data Fig. 2 [file 44319_2024_365_MOESM4_ESM.zip › Figure 2/2B/2B, pH 7.4, ALFA-PRF.tif]

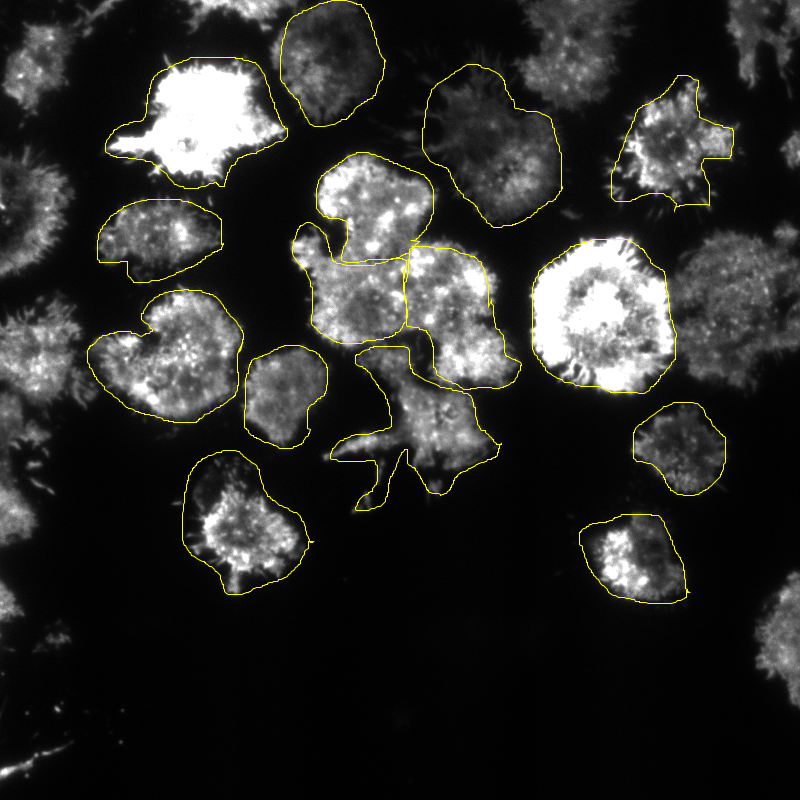

Supplement: Supplementary file 4 — Source data Fig. 2 [file 44319_2024_365_MOESM4_ESM.zip › Figure 2/2B/2B, pH 7.4, F-actin.tif]

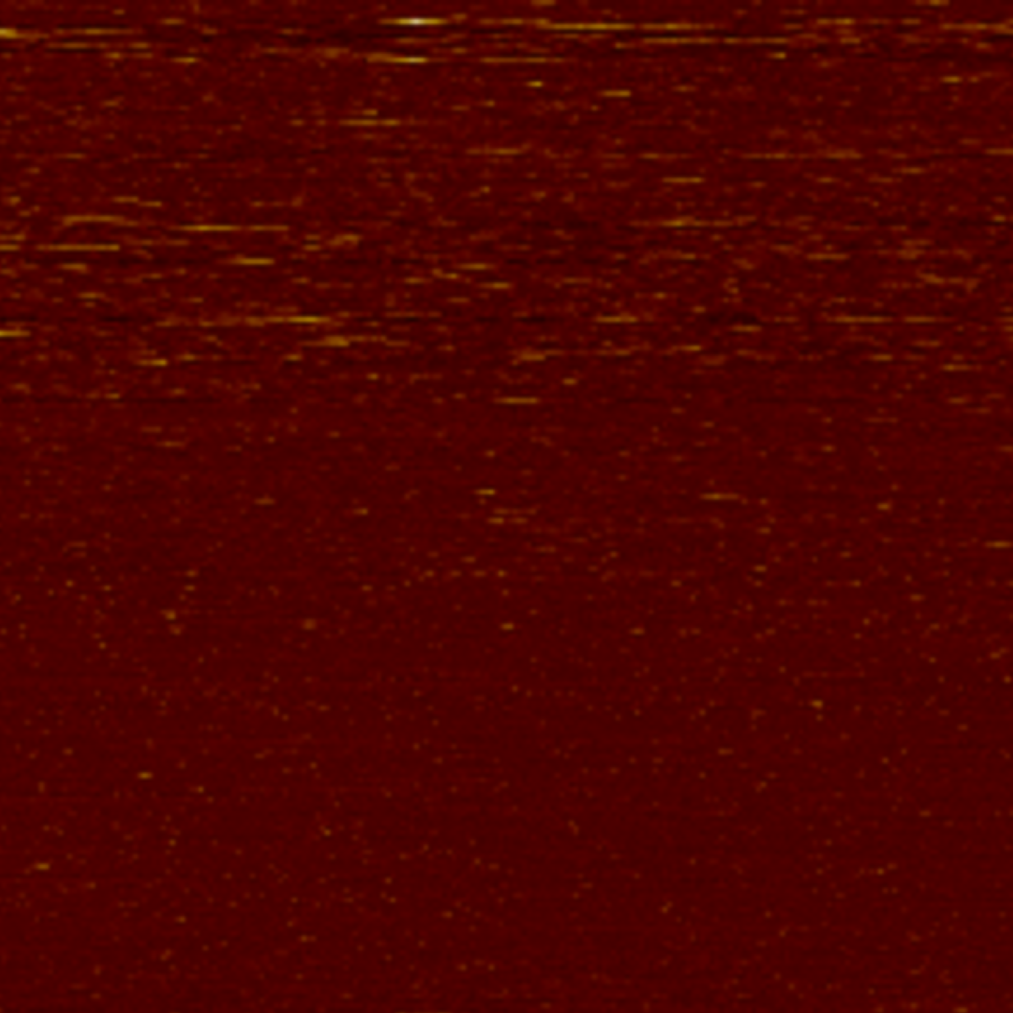

Supplement: Supplementary file 5 — Source data Fig. 3 [file 44319_2024_365_MOESM5_ESM.zip › Figure 3/3B/3B, pH 5.png]

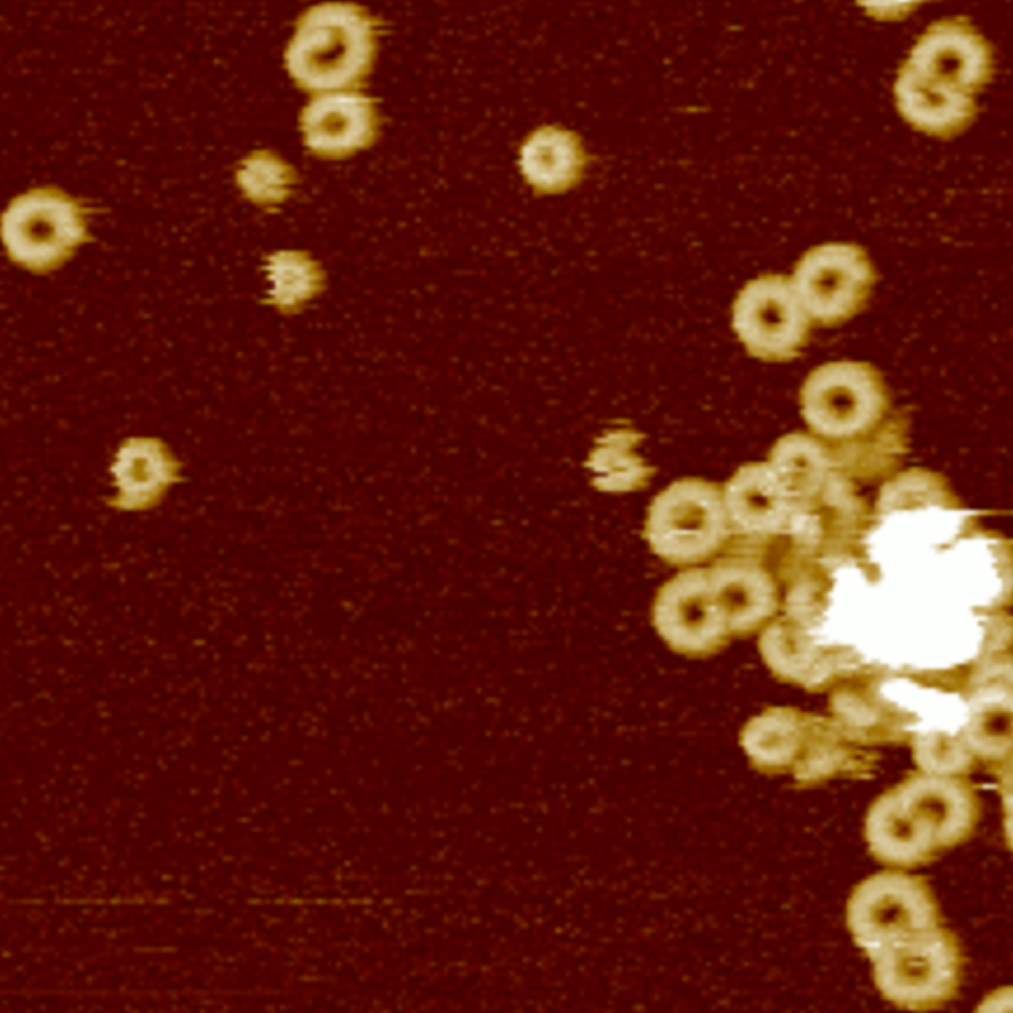

Supplement: Supplementary file 5 — Source data Fig. 3 [file 44319_2024_365_MOESM5_ESM.zip › Figure 3/3B/3B, pH 6.5.png]

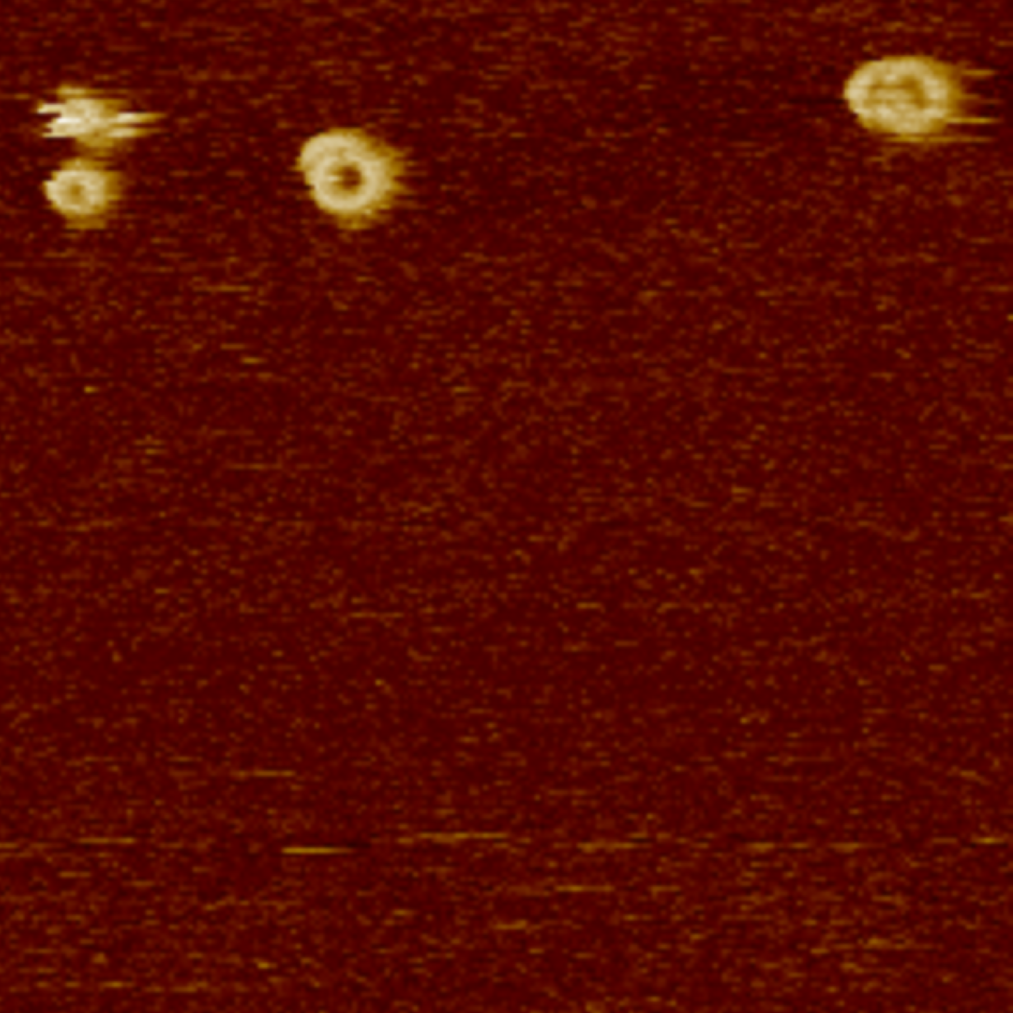

Supplement: Supplementary file 5 — Source data Fig. 3 [file 44319_2024_365_MOESM5_ESM.zip › Figure 3/3B/3B, pH 6.png]

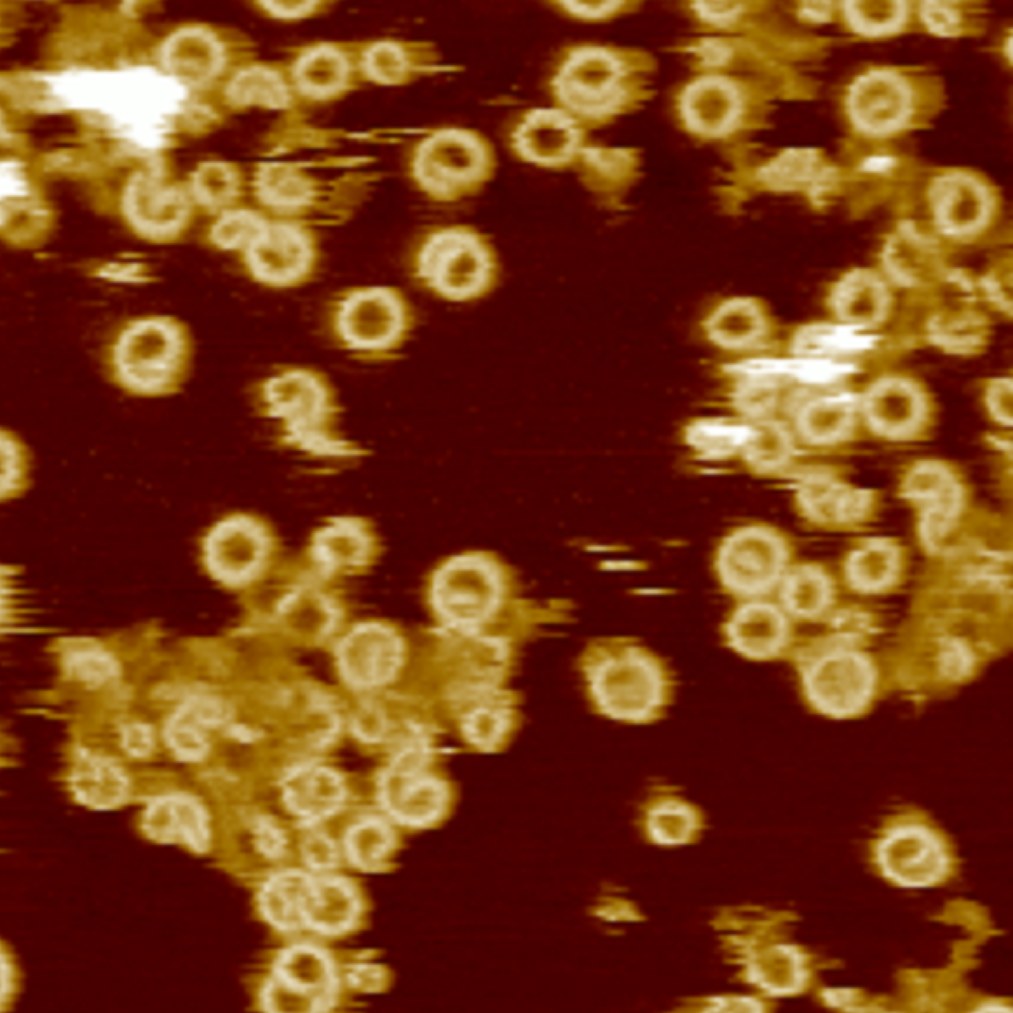

Supplement: Supplementary file 5 — Source data Fig. 3 [file 44319_2024_365_MOESM5_ESM.zip › Figure 3/3B/3B, pH 7.4.png]

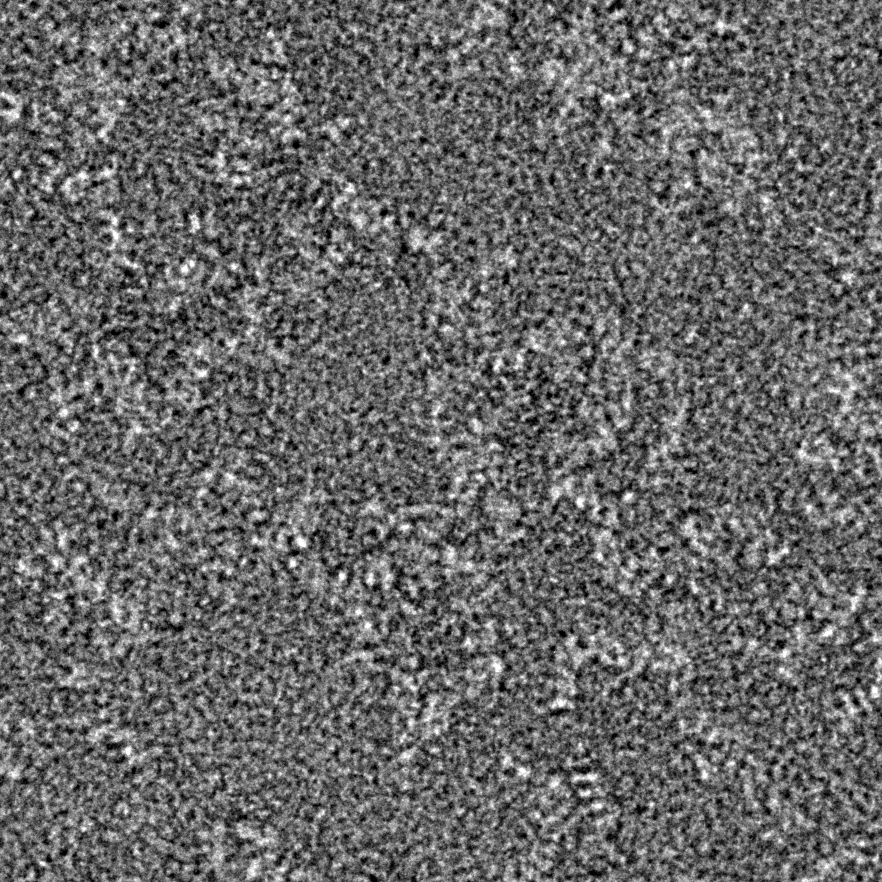

Supplement: Supplementary file 6 — Source data Fig. 4 [file 44319_2024_365_MOESM6_ESM.zip › Figure 4/4E/4E, pH 6.png]

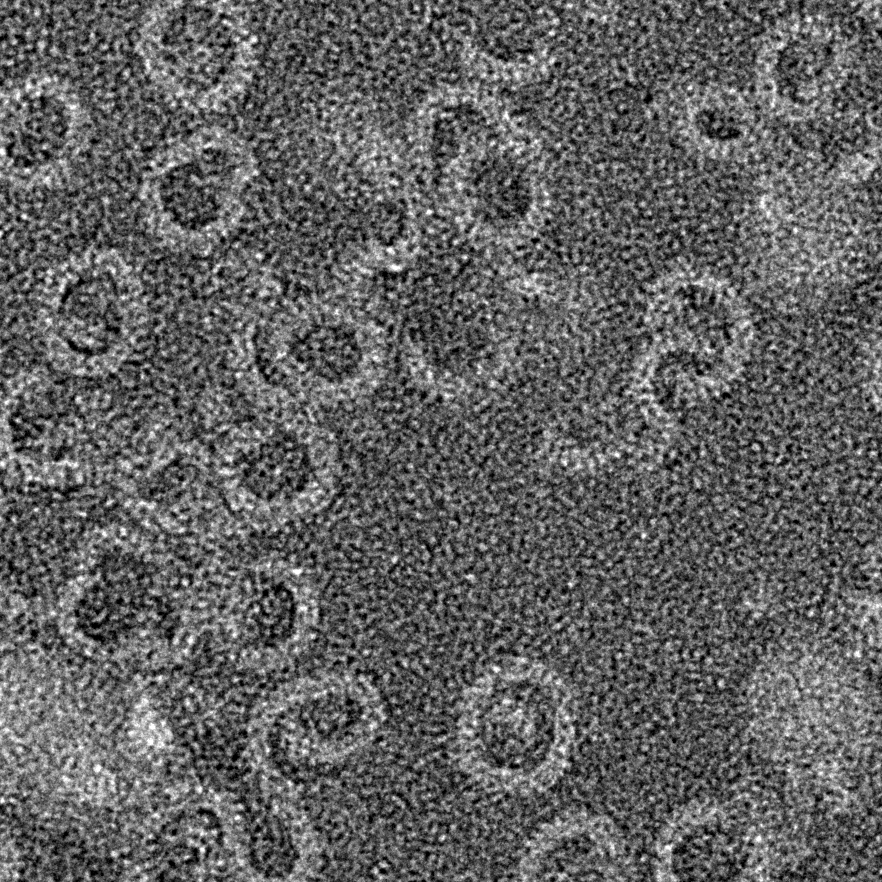

Supplement: Supplementary file 6 — Source data Fig. 4 [file 44319_2024_365_MOESM6_ESM.zip › Figure 4/4E/4E, pH 7.5.png]
