# Supplementary material for: Acidic pH can attenuate immune killing through inactivation of perforin
Source: EMBO Rep. 2025 Jan 9;26(4):929–47. doi: 10.1038/s44319-024-00365-6 (PMC11850619; doi:10.1038/s44319-024-00365-6)
Supplement: Supplementary file 7 — Expanded View Figures [file 44319_2024_365_MOESM7_ESM.pdf]

Expanded View Figures

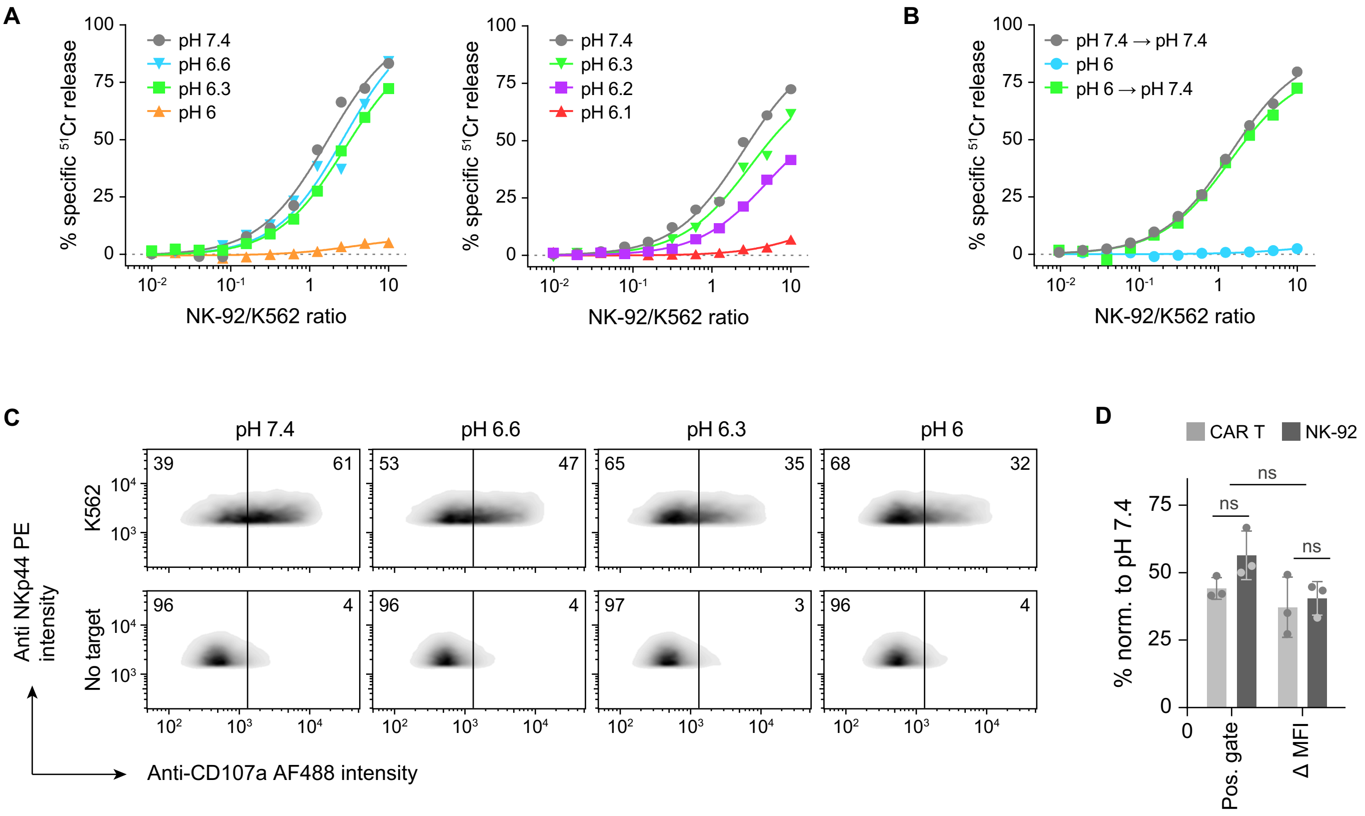

Figure EV1. NK-92 cells degranulate but do not kill K562 target cells at acidic pH.

(**A**) Killing assays performed at different pH outline a marked reduction of killing between pH 6.3–6. (**B**) Immune killing of cells incubated for 4 h at pH 6 and 37 °C was fully restored after overnight incubation at neutral pH, 37 °C. (**C**) Degranulation of NK-92 cells after mixing with K562 target cells at different pH shows a reduction, but not abrogation of degranulation. Of note, degranulation at pH 6.3 and 6 was almost identical, while the killing capacity was abolished (see (**A**)). (**D**) Degranulation at pH 6 compared to degranulation at neutral pH of CAR T (as in Fig. 1C) and NK-92 cells (as in (**C**)). Shown are degranulation levels estimated by positivity gate (pos. gate, as in (**C**)) or by increase of geometric mean fluorescence intensity compared to control samples without target cells (Δ MFI). Data information: (**A**) shows $n = 2$ biological replicates in separate plots. (**B**, **C**) shows data from single experiments, $n = 1$. In (**D**), data presents mean ± SD from $n = 3$ biological replicates. No significant differences between different conditions were found using Kolmogorov-Smirnov tests, ns, not significant, $P = 0.1$ (pos. gate), $P = 1.0$ (Δ MFI), $P = 0.5$ (pos. gate pooled vs. Δ MFI pooled).

 © The Author(s)

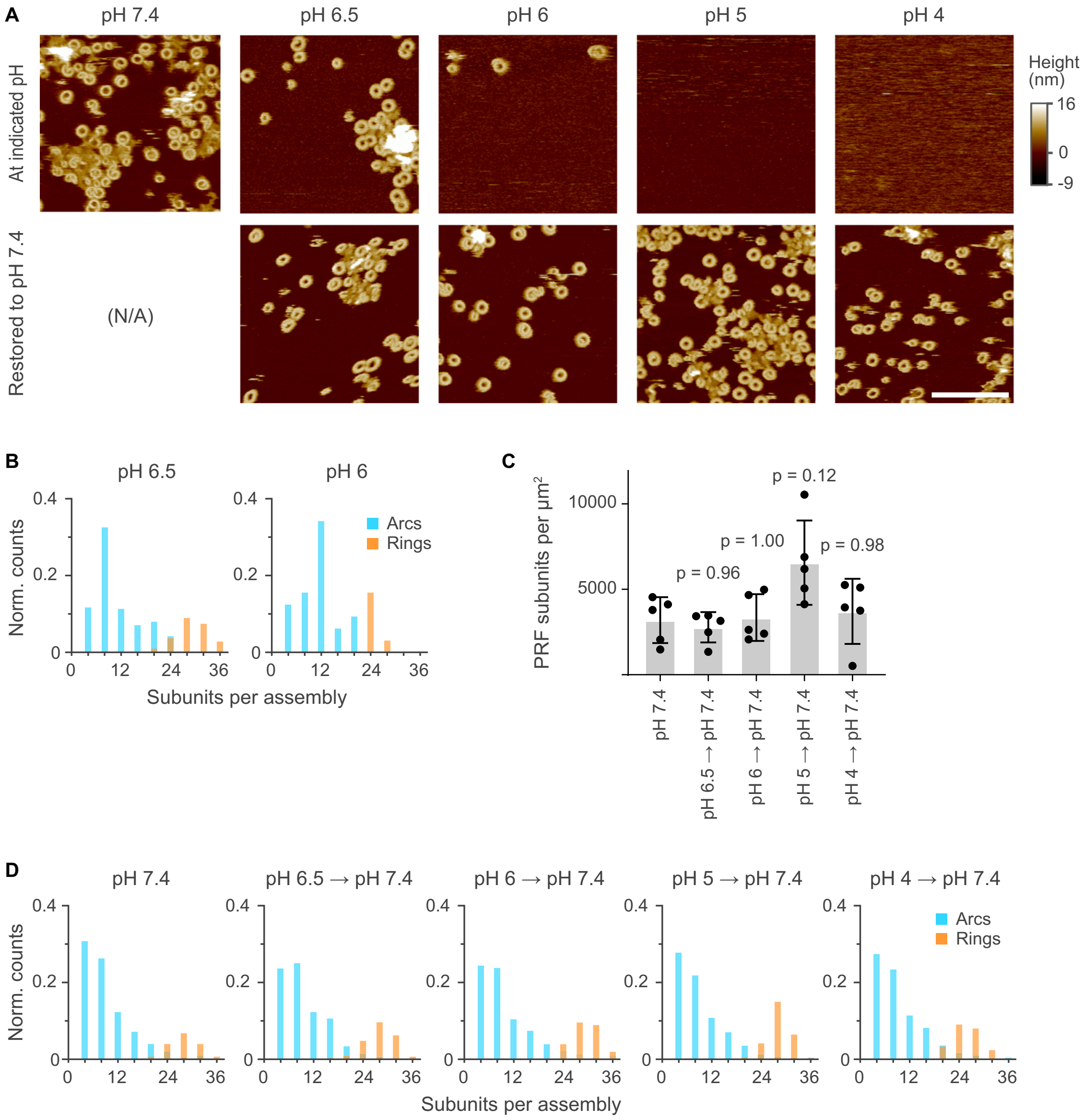

Figure EV2. AFM detection of WT-PRF pores at different pH before and after neutralisation.

(**A**) The top row of AFM images exemplifies pH-dependent WT-PRF pore formation. The samples were incubated with WT-PRF for 5 min at 37 °C and at indicated pH levels and subsequently imaged at room temperature for up to 1 h. The bottom row shows the same samples after restoring the pH by washing with pH 7.4 buffer and incubating for 15 min at 37 °C. The number of pores is visibly reduced at pH 6 before restoration and is absent in samples incubated at pH 5 and 4. After restoration of pH, WT-PRF pores emerge again in all samples, irrespective of prior pH. N/A, not assessed. (**B**) At pH 6.5 and pH 6, a reduced number of pores are formed. Their size distributions show arc- and ring-shaped assemblies. (**C**) An evaluation of protein densities on the sample surface after restoring pH from acidic conditions shows no significant deviations from samples prepared at neutral pH, showing that protein binding is not affected by pH. (**D**) Assembly size distributions of WT-PRF pores after their restoration to neutral pH. The shape of these distributions appears conserved across all pH levels. They also resemble distributions obtained on assemblies formed before the restoration of pH, shown in (**B**), overall indicating that the mechanism WT-PRF is not fundamentally altered by pH and can recommence its pore formation using the same oligomerization pathway as in neutral pH. The data shown in (**B**) and (**D**) was collected from 5 images taken across the sample surface, covering a total area of 1.5 µm^2 each. Data information: (**A**) scale bar, 200 nm. Distributions in (**B**) are shown from a total of $n = 212$ (pH 6.5) and $n = 32$ (pH 6) assemblies. In (**C**), Welch's ANOVA test with post hoc Dunnett's test detected no significant differences of protein densities of restored samples compared to pH 7.4; exact p values indicated atop each bar. Distributions in (**D**) are shown from a total of $n = 576$ (pH 7.4), $n = 417$ (pH 6.5 → pH 7.4), $n = 464$ (pH 6 → pH 7.4), $n = 965$ (pH 5 → pH 7.4), and $n = 593$ (pH 4 → pH 7.4) assemblies.

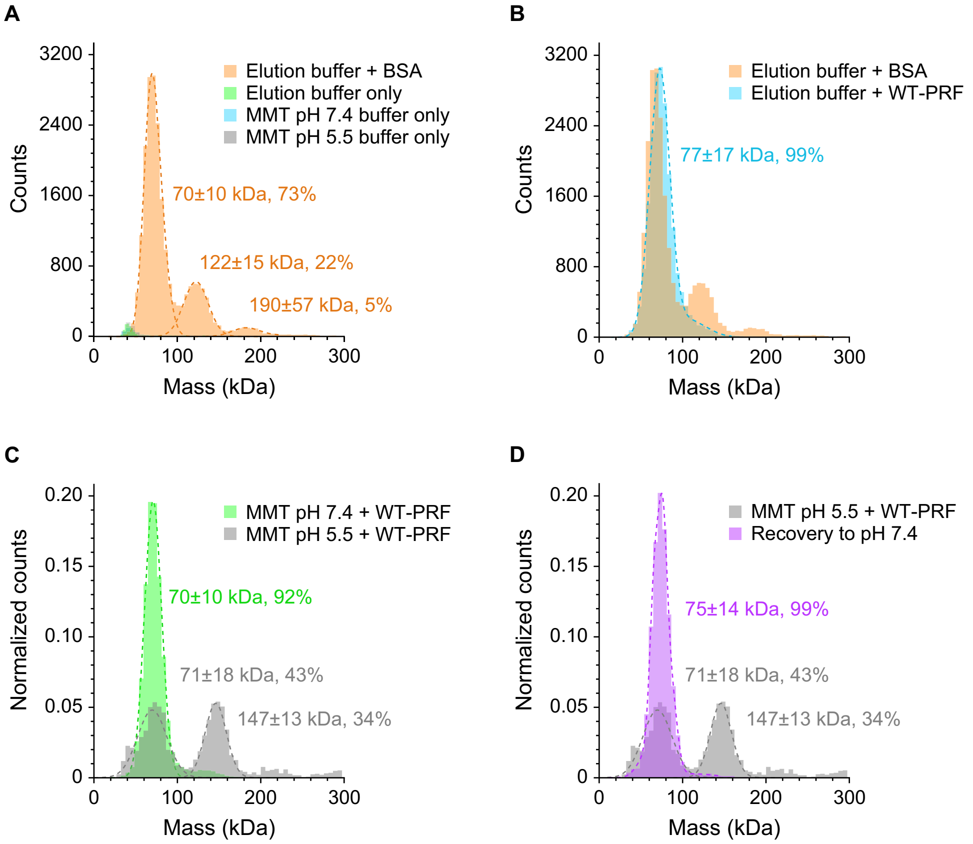

Figure EV3. Mass photometry of WT-PRF in solution.

(**A**) The prominent monomer and dimer peaks of bovine serum albumin (BSA, orange) in elution buffer were used to calibrate the mass photometry system. The buffers alone (green, blue, grey) produced a minor signal at around 40 kDa, which we interpret as noise at the lower boundary of the detection range. (**B**) Wild-type murine perforin (WT-PRF, blue) in elution buffer produced a single peak overlapping with the BSA (orange) monomer signal and estimated at 77 kDa molecular weight, approximately corresponding to the molecular mass of monomeric WT-PRF. (**C**) A similar singular peak for monomeric WT-PRF is observed in MMT buffer at pH 7.4 (green), whereas at pH 5.5, two peaks with molecular masses approximately corresponding to monomeric and dimeric WT-PRF are dominant (grey). (**D**) Dimeric WT-PRF found in MMT at pH 5.5 (grey, reproduced from (**C**)) disassembled into monomers upon restoring the buffer to pH 7.4 (purple).

 © The Author(s)

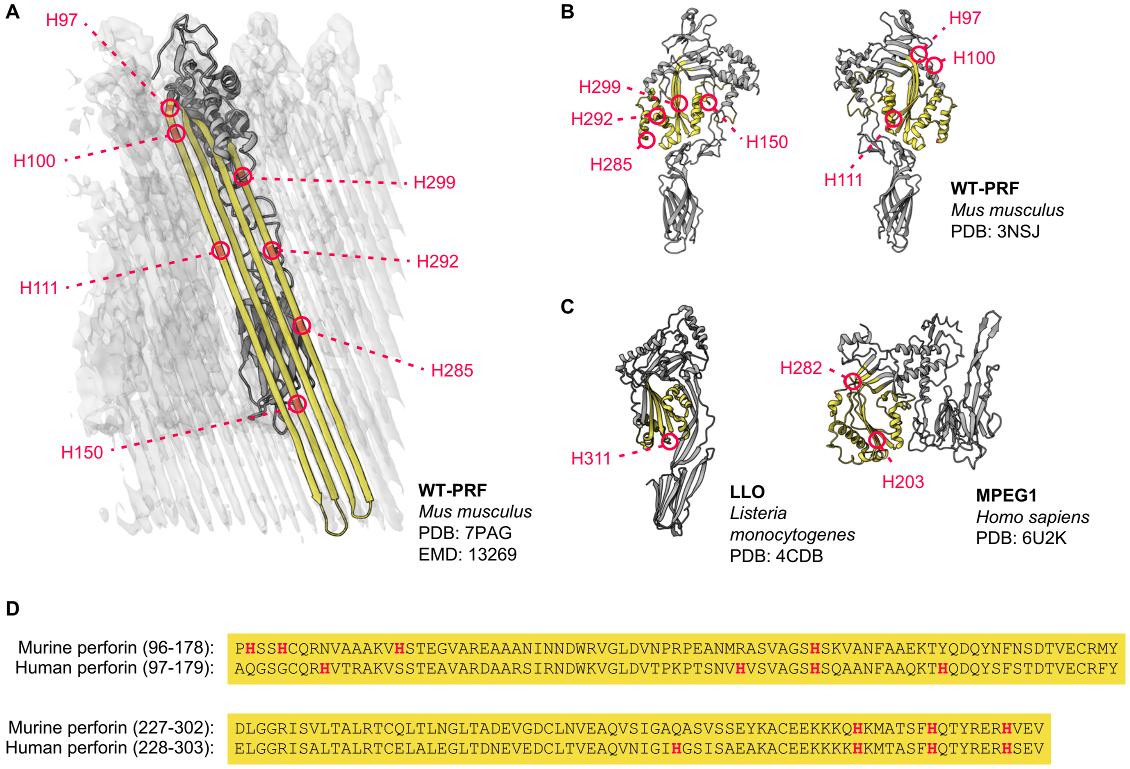

Figure EV4. Histidine residues at transmembrane β-hairpin interfaces of perforin.

(**A**) Cartoon of a murine WT-PRF subunit in the pore state, overlayed with a section of an electron density map of the perforin pore (grey) (Ivanova et al, 2022; Data ref: Ivanova et al, 2022a; Data ref: Ivanova et al, 2022b). The transmembrane β-barrel (yellow) motif of WT-PRF is seamed with numerous histidine residues (red). (**B**) The same histidine residues as in (**A**) highlighted in the soluble monomer structure of WT-PRF, shown from two sides (Law et al, 2010b; Data ref: Law et al, 2010b). (**C**) By comparison, the acid activated pore-forming proteins listeriolysin O (LLO) and macrophage expressed gene 1 (MPEG1) contain fewer histidine residues (red) in the corresponding regions (yellow, green). Protein models were generated from the RCSB protein database using the indicated model accessions (Köster et al, 2014; Pang et al, 2019; Data ref: Köster and Yildiz, 2014; Data ref: Pang and Bayly-Jones, 2019). (**D**) Comparison between wild-type murine and human perforin amino acid sequence for the β-barrel motif. The sequences were retrieved from UniProt under accessions P10820 and P14222, respectively, and aligned. Histidine residues are highlighted in red and are similarly abundant in both murine and human PRF, but not necessarily conserved.
